# Psychophysical and Social Functioning of Patients with Chronic Obstructive Lung Disease and Depression

**DOI:** 10.3390/ejihpe13120196

**Published:** 2023-12-04

**Authors:** Ivana Jelić, Goran Mihajlović, Miloš Ratinac, Katarina Mihajlović, Sara Mijailović, Ivan Čekerevac

**Affiliations:** 1 Faculty of Medical Sciences, University of Kragujevac, Svetozara Markovića 69, 34000 Kragujevac, Serbia; drgoranmih@gmail.com; 2 Clinic of Psychiatry, University Clinical Center Kragujevac, 34000 Kragujevac, Serbia; 3 Clinic of Pulmonology, University Clinical Center Kragujevac, 34000 Kragujevac, Serbia; milos.ratinac@gmail.com (M.R.); icekerevac@gmail.com (I.Č.); 4 Department of Pharmacy, Faculty of Medical Sciences, University of Kragujevac, 34000 Kragujevac, Serbia; katarina.radonjic@medf.kg.ac.rs; 5 Department of Medical Statistics and Informatics, Faculty of Medical Sciences, University of Kragujevac, 34000 Kragujevac, Serbia; saramijailovic212@gmail.com; 6 Department of Internal Medicine, Faculty of Medical Sciences, University of Kragujevac, 34000 Kragujevac, Serbia

**Keywords:** antidepressant therapy, chronic obstructive lung disease, depression, dyspnea, psychophysical aspect, social functioning, somatic symptoms, quality of life

## Abstract

The relationship between depression and chronic obstructive pulmonary disease (COPD) is not fully understood. The comorbidity rate ranges from 18 to 84%, and depression is closely related to chronic inflammation, which affects how patients and the people around them perceive their condition. This study aims to examine the relationship between the psychophysical and social functioning of COPD patients who have been diagnosed with depression and the therapeutic benefits of selective serotonin reuptake inhibitors (SSRIs). This prospective study enrolled primary care patients diagnosed with COPD and depression. The entire period of this research was 3 years. The research was conducted at the Primary Health Center, Kragujevac, Serbia, in 87 patients for 8 weeks. The Hamilton Depression Scale (HAM-D) and Questionnaire for Quality of Life and Life Satisfaction Short Form (Q-LES-Q-SF) were used for psychiatric assessment. A positive correlation was found between the psychophysical and social functioning of the participants after 8 weeks of treatment with SSRIs. Based on the value of the phi correlation coefficient (phi = 0.5) obtained using the χ^2^ test, a large influence was observed in terms of life satisfaction and physical health (*p* < 0.05). In terms of physical functioning by gender, based on the value of the Pearson’s coefficient (r) obtained with the χ^2^ test, it was shown that physical functioning was superior in the female respondents (*p* < 0.05). Treatment was found to improve depression in COPD after 8 weeks of therapy.

## 1. Introduction

Chronic obstructive pulmonary disease (COPD) is characterized by a progressive and persistent limitation of flow in the airways. This limitation is the result of a chronic inflammatory response in the airways that is triggered by the occasional exposure of the lungs to harmful gases and particles. Smoking, external, internal, and occupational air pollution, infections, and genetics are the main risk factors for the condition [1]. Worldwide, it is the third most common cause of mortality, accounting for over 3 million fatalities in 2019, and it ranks sixth among all causes of loss of disability-adjusted life years (DALYs), accounting for over 74 million DALYs in the same year [2,3]. 

COPD is associated with numerous comorbidities that increase overall mortality. Depression and anxiety are psychiatric conditions that are often associated with a lower survival rate and impaired social functioning in chronic diseases such as COPD, and the mechanism of their development is multifactorial [4]. Several inflammatory cytokines are elevated in the circulation and bronchoalveolar lavage of such patients, such as TNFα, IL-6, IL-8, CRP, fibrinogen, and serum amyloid A. Their transition into systemic circulation causes systemic inflammation that can initiate or worsen already existing comorbidities [4,5]. 

It is important to highlight that dyspnea is the most common symptom in COPD patients. Research explains that many patients adopt a sedentary lifestyle to prevent exertional dyspnea, which, over time, causes significant skeletal muscle weakness, social isolation, and detrimental psychological effects [6]. 

Dyspnea is strongly impacted by emotions and psychosocial factors, including personality, anxiety, and depression. Under the same circumstances, those with psychiatric problems will perceive their dyspnea as more acute and upsetting than people without psychiatric comorbidities [7]. 

Clinically significant dyspnea (designated as, according to the Modified Medical Research Council Dyspnea Scale, mMRC ≥ 2) is associated with depression and anxiety [8]. Exposure to cigarette smoke, inactivity, social isolation, multiple bouts of dyspnea, chronic hypoxia, female gender, and loneliness are some of the causes of this association [9,10,11]. Research shows that the prevalence of depression in patients with COPD is 26% [12]. This risk is even greater in patients who are on long-term oxygen therapy at home [13]. 

Reduced sexual activity, dependence on others for personal care, loss of interest in future events, retreat, and a general sense of helplessness are potential effects of anxiety and depression [14]. Depression is often untreated or insufficiently treated in this group of patients, which worsens their health status and quality of life and increases overall mortality [15]. The subset of COPD patients who suffer from depression has a much higher risk of exacerbations; hence, the annual expenditures for treating these patients are significantly higher [16]. 

COPD patients who suffer from mild depression can benefit from cognitive behavioral therapy (CBT) and pulmonary rehabilitation, while those with persistent or severe depression can undergo treatment with medication [17]. There are numerous difficulties in the diagnosis of depression in patients with COPD, both by patients (lack of knowledge about depression, stigmatization of the mentally ill, resistance to reveal symptoms when asked about them, justifying symptoms of mood disorders by the existence of physical symptoms such as fatigue, etc.), and by doctors (lack of knowledge about depression in COPD, short examination period, lack of time to educate and counsel patients about depression, lack of skills to perform psychiatric assessment, etc.) [18]. Currently, there are no evidence-based recommendations for the management of COPD-related depression and the administration of antidepressants in this patient population [19]. SSRIs (selective serotonin reuptake inhibitors) are medications commonly used in the treatment of depression. A number of studies have suggested that SSRIs may also have a beneficial effect on the symptoms of anxiety, depression, and mood disturbances experienced by patients with COPD. These medications may help improve overall well-being, reduce anxiety and depression, and enhance quality of life in COPD patients [18,19,20].

Previous research has shown that the correct assessment of the symptoms of depression and lowered quality of life leads to an easier choice of priorities in planning therapeutic procedures and faster, enhanced communication between doctors and patients, as well as an easier diagnosis of a patient’s potential problems. It is also the most accurate way to find out how realistic a patient’s expectations of treatment are, the measures to monitor changes during treatment, and the quality of care provided to a patient, as well as the entire treatment outcome [20,21].

The aim of this study was to determine the correlation between the psychophysical and social functioning of patients with COPD who have been diagnosed with depressive disorder and to investigate the effects of therapy with selective serotonin reuptake inhibitors (SSRIs). In order to do so, the methods used are presented in the following section.

## 2. Materials and Methods

### 2.1. Participants

Primary care patients with COPD and depression were included in this prospective trial. Three years of research were conducted at the Primary Health Center in Kragujevac, Serbia, during the period October 2016–December 2019. There were 87 patients in the study population. To take part, every patient signed a written consent form.

At the very beginning of the study, the total number of identified patients with COPD was 604. All patients were invited to participate for the entire study period, and 522 agreed to participate. The patients who agreed to participate in the study provided written informed consent. Among them, 19 patients were excluded because of the presence of another mental disorder apart from depression. In addition, somatic exclusion criteria were used, which caused the further exclusion of patients from the study (i.e., type 1 diabetes mellitus: 8; malignant diseases: 0; ventricular arrhythmias: 2; ischemic heart disease: 4; and chronic renal or liver failure: 2). Finally, 3 patients were excluded after the initiation of SSRIs due to the adverse effect of the antidepressants, and 1 patient was lost to follow-up. Because of the deterioration of patients’ somatic disease, hospitalization, and additional illnesses that appeared during the research process and were included as eliminative factors, 396 of the 522 initial participants were excluded from the study. According to these procedures, the final sample of patients who completed the study was 87 subjects. No specific programs (i.e., pulmonary rehabilitation, smoking cessation, dietician consultation, drug modification, or psychological support) were applied during the study.

### 2.2. Instruments

#### 2.2.1. Pulmonary Obstruction and Dyspnea

The clinical diagnosis of COPD was established on the basis of pulmonary function tests, according to the Global Initiative for COPD [22]. Dyspnea was graded using a modified British Medical Research Council (mMRC) scale. The mMRC scale contains 4 categories, and each of them defines the degree to which breathlessness can affect a person’s ability to perform daily activities; scores ≥ 1 correlate with increased dyspnea. This scale, in which 0 represents the lowest grade (i.e., non-endangered shortness of breath) and 4 represents the most endangered shortness of breath, is a self-assessment tool for measuring the degree of breathlessness during daily activities [23,24]. Based on the mMRC scale, patients with COPD are categorized into three groups [25]. Patients in the first group are those who have mild lung disease (level 1—mMRC 1), those in the second group have moderate lung illness (level 2—mMRC 2 and mMRC 3), and those in the third group have severe and very severe lung disease (level 3—mMRC 4).

#### 2.2.2. Depression

A clinical diagnosis of depression was established by psychiatrists within the framework of routine healthcare. Symptoms of depression were assessed using the Patient Health Questionnaire-9 (PHQ-9) screening test [26] and the Hamilton Depression Scale (HAM-D) [27]. 

The PHQ-9 is a self-report questionnaire for patients that is used to evaluate the most common psychiatric disorders at the level of primary care. The PHQ-9 is an abbreviated version of the PHQ and applies only to patients with a depressive disorder because it measures the 9 criteria required for a diagnosis of a depressive disorder. 

The HAM-D [27,28] was employed to evaluate the severity of the symptoms of depression. It is considered very reliable and valid, and it is one of the most used scales in research. This scale can be applied to psychiatric and non-psychiatric patients, as well as the healthy population. The HAM-D consists of 17 questions and ranks the answers on a 4-point scale from 0 to 3, whereby higher values on the scale indicate the presence of more intensively expressed depressive symptoms. The total score is calculated by simply adding up all of the answers, and its value ranges from 0 to 63. To grade the severity of depression, patients were divided into groups according to their HAM score as follows: values from 0 to 13—without depression (group 0); values from 14 to 19—mild depression (group 1); values from 20 to 28—moderate depression (group 2); and values from 29 to 63—expressed depression (group 3). 

#### 2.2.3. Quality of Life

The Questionnaire for Quality of Life and Life Satisfaction Short Form (Q-LES-Q-SF) [29] was used to assess the quality of life. This questionnaire represents a psychometrically correct and effective method of measurement and enables the regulation of the quality of life in different diseases and populations. The Q-LES-Q-SF was used according to the following psychophysical and social scales: bad, average, and good. Each of the fourteen items was rated on a 5-point scale indicating the degree of enjoyment or satisfaction during the past week. The total score of all fourteen items (ranging from 14 to 70) was calculated and expressed as a percentage (1–100) of the maximum total score. The minimum value of the score is 0, and the maximum is 100. The higher the score, the better the quality of life, i.e., indicating greater satisfaction. 

All patients received SSRI therapy, and they were monitored for 8 weeks. A psychiatrist checked on the patients after 8 weeks, reevaluating their mental health and degree of depression (using the HAM-D scale).

### 2.3. Ethics Statements

After thoroughly explaining the study to the participants in a language they could understand, which mostly avoided the use of complex medical terms, their informed consent was obtained. No biological sample was taken. Confidentiality was preserved. This study was approved by the Ethics Committee of the Health Center, Kragujevac, Serbia.

### 2.4. Statistical Analysis

By applying the mentioned parameters and using an appropriate statistical analysis program, *t*-tests were conducted for two independent samples, with an alpha of 0.05 and a power of the study of 0.8, for the ratio of the subjects in the groups, and bearing in mind that the design of the study envisaged three groups of patients, 87 respondents were determined for the total study population. The data analyses were performed using the Statistical Package for Social Science (SPSS) (Version 19.0). The numerical data are shown as the mean and standard deviation (SD) or median and interquartile range (IQR); the categorical data are shown as the number and percentage of the total. Categorical variables were analyzed using the χ^2^-test. The Mann–Whitney U test was used to determine the difference between two independent groups, and the Wilcoxon signed-rank test was used to compare the difference before and after the SSRI therapy. Internal consistency was determined using Cronbach’s alpha. A *p*-value of 0.05 or less is considered significant. 

## 3. Results

### 3.1. Sample Characteristics

The study sample consisted of 87 participants, of which 57 (65.52%) were women and 30 (34.48%) were men. The subjects’ average age was 48.84 ± 7.43 years. The age difference between genders was statistically significant (*p* = 0.033). The mMRC analysis showed that 37.93% (N = 33) of the subjects had mild (level 1), 51.72% (N = 45) had moderate (average/severe) (level 2), and 10.53% (N = 9) had severe and very severe dyspnea (level 3). Table 1 represents the demographic, clinical, and social characteristics of the patients in the total sample and in relation to gender.

The labels HAM-D1 and HAM-D2 were used to differentiate the HAM-D scores before and after SSRI therapy, respectively. According to a comparison of belonging to groups in relation to the HAM-D1 and HAM-D2 values, there was a statistically significant improvement (χ^2^ = 131.322, Cramer’s V = 0.946, *p* < 0.001) after 8 weeks of therapy. At the beginning of the study (before 8 weeks of medication), there was a statistically significant difference between the level of depression and the stage of COPD in patients. After 8 weeks of medication, the majority of patients had positive outcomes as measured by the HAM-D2, regardless of the severity of the underlying disease. Results revealed a significant relationship between the mMRC and degree of depression before (χ^2^ = 35.241, Cramer’s V = 0.482, *p* < 0.001) and after 8 weeks of depressive disorder treatment (χ^2^ = 36.011, Cramer’s V = 0.474, *p* < 0.001). The internal consistency for HAM-D1 was 0.939, and for HAM-D2, it was 0.889, indicating good internal consistency.

### 3.2. Psychophysical and Social Functioning of Subjects in the Study

#### 3.2.1. Quality of Life before and after Medication

The level of dyspnea and the difference in the Q-LES-Q-SF score were significantly correlated, showing that, on average, patients had a remarkable improvement in psychophysical and social functioning. After 8 weeks of SSRI therapy, there was a statistically significant improvement in the Q-LES-Q-SF score (Table 2). The internal consistency for Q-LES-Q-SF was 0.706 before and 0.667 after SSRI therapy.

**Figure 1:**
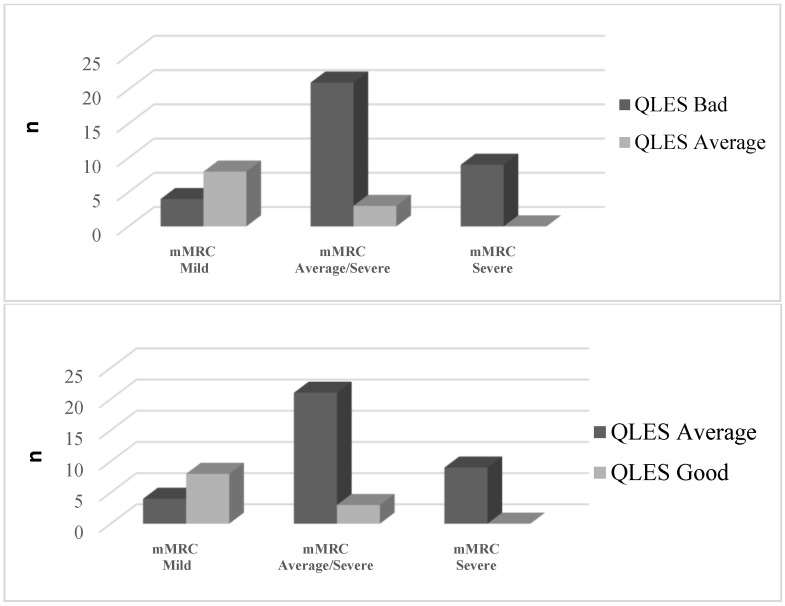
Frequency (n) of male patients in relation to the quality of life (divided into bad, average, good groups) and the level of mMRC (mild, average/severe, and severe) before (**up**) and after (**down**) 8 weeks of medication.

**Figure 2:**
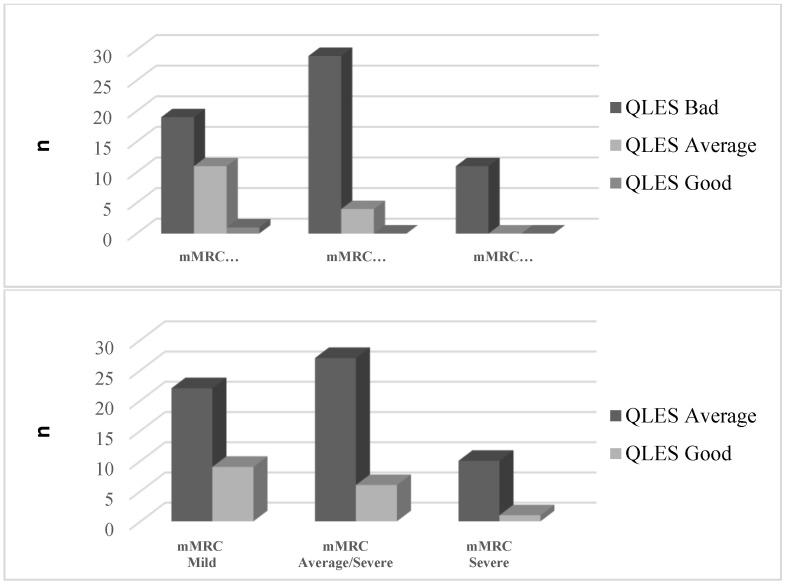
Frequency (n) of female patients in relation to quality of life (divided into bad, average, good groups) and level of mMRC (mild, average/severe, and severe) before (**up**) and after (**down**) 8 weeks of medication.

When individuals were treated with SSRIs, multiple analyses revealed a statistically significant difference among the Q-LES-Q-SF scores (*p* = 0.01) in terms of the subjects’ quality of life and total life satisfaction. Furthermore, the obtained difference in the Q-LES-Q-SF scores was significantly higher in patients with very severe dyspnea than in moderate (average/severe) and mild dyspnea levels, based on mMRC values (F = 5.680, η^2^ = 0.110, *p* < 0.001). As for gender, the difference in the scores was equally distributed across groups (F = 0.691, Cohen’s d = 0.118, *p* = 0.270). The overall quality of life of both the male and female participants improved after medication, as shown in Figure 1 and Figure 2, respectively. All patients in the QLES bad stage progressed from QLES bad to QLES average after 8 weeks of medication. After SSRI therapy, more than half of patients were in the QLES average stage.

#### 3.2.2. Level of Depression and Quality of Life

According to the results of the analysis, differences in the HAM and Q-LES-Q-SF scores were found. Namely, after 8 weeks of SSRI therapy, there were statistically significant improvements in the HAM-D1 (*p* < 0.001) and Q-LES-Q-SF (*p* < 0.001) scores (Table 3). 

#### 3.2.3. Improvements in Single Domains of Quality of Life

There was a statistically significant improvement in the quality of life parameters before and after 8 weeks of application of SSRIs, except in the case of relationship to family (Table 4). There was a statistically significant correlation between age and difference in Q-LES-Q-SF score (r = 0.355, *p* < 0.001), indicating that older patients had a more significant improvement in quality of life.

When the quality of life parameters were observed, women had statistically significantly higher life satisfaction both before (U = −3.225, η^2^ = 0.676, *p* = 0.027) and after the use of therapy (U = −2.963, η^2^ = 0.675, *p* = 0.036) (Table 5). Other parameters in both measurement moments were not related to gender (*p* > 0.05). 

A comparison of the quality of life of respondents with COPD and depressive disorder before and after the introduction of antidepressant therapy shows a statistically significant difference (χ^2^ = 24.620, *p* < 0.05). Namely, after 8 weeks of treatment for depressive disorder, no respondent rated their quality of life as bad. Good quality of life was rated by respondents who either did not have or had a mild degree of depression. All other respondents, regardless of the degree of depressive disorder, believed that their quality of life was average (i.e., neither good nor bad).

## 4. Discussion

The results of this study confirmed that there is a positive correlation between the incidence of co-occurring depression and the psychophysical and social functioning of patients. The relationship between symptoms and quality of life is a key issue in the treatment of mood disorders. Depressive symptoms indirectly affect the environment and social quality of life and are associated with various interrelated factors, such as the ability to work, employment status, living situation, and the quality of socially effective relationships, which are allied to their relationship with people. These outcomes partially correlate with a study by Hobart et al., in particular with the claim that medicament therapy impacts depression symptom reduction [30]. This result has also been proven in population studies with this disease, such as in studies by Di Marco et al. [10], in which the authors found that women are at greater risk because many of them, in addition to doing housework, tend to live alone or care for the elderly. For example, the research of Di Marco et al. [10] found that women with COPD, in addition to being more prone to depression than men, also showed worse symptoms in variables related to quality of life. According to the research results of Morton et al. [31], the quality of life in patients with COPD and comorbid depression improved with adjunctive therapy. After this therapy, a positive change in the physical and psychological quality of life was shown. In other words, there was an improvement in functioning in all domains of quality of life, which correlates with the results obtained in our study.

Numerous different aspects of quality of life were considered in the results, and the interaction between these conditions had a serious impact on how patients evaluate their quality of life. An examination of patients’ quality of life revealed that, while physical health was one area in which gender significantly influenced the assessment of the quality of life and overall perception of personal condition, gender has little bearing on patients’ general health, emotional issues, or vitality. According to different studies [32,33], gender plays a crucial role, with women recognizing their illnesses less coherently than men. Accordingly, scientists who study the prevalence, causes, and development of depressive disorders show a great interest in researching gender differences [1], but they have different opinions. Some claim that psycho-pathological phenomena are more common in women; according to others, they are more present in men. There are also those who believe that both sexes face the same experiences. In our study, as for gender, the difference in the scores was equally distributed across groups. The overall quality of life of both the male and female participants improved after 8 weeks of application of SSRI therapy.

In terms of physical health, research reveals that younger patients are more emotionally attached to their sickness than older patients, who ascribe more symptoms to their illness [25]. Variations in connection to the subject’s sex can be seen in the psychophysical and social functioning of subjects with COPD before and after the introduction of antidepressant medication. Men are more likely than women to rate their level of life satisfaction as bad while having moderate dyspnea. In this study, women had higher life satisfaction both before and after receiving therapy when psychophysical and social functioning measures were examined. Other factors were unrelated to gender in any of the measurement instances. These findings are consistent with earlier research [34] and demonstrate that there are gender differences in how people perceive the effects of their disease, which has an impact on their level of overall life satisfaction. 

The impact of depression is significant not only on the sufferer but also on their families and environment [33,35]. Depressed patients with chronic illness(es) tend to be “sicker” than those without chronic illness(es) [35,36], which often impacts their quality of life. In fact, according to the life satisfaction and quality of life scores of patients with COPD and depression, this study shows that their relationship with their family improved after 8 weeks of therapy, but it was not of statistical significance.

This study found that there was a decreased capacity for adaptation, particularly in terms of how the subjects interacted with themselves. Therefore, it is confirmed that it is necessary to use therapeutic guidelines for each patient and each disease separately [11]. When appropriate therapy protocols are used for individuals with comorbid depression in this context, all drugs should be considered for rational drug delivery. To enhance patients’ quality of life, a holistic approach should be used throughout [19]. 

Depression in COPD reduces quality of life [37], which was confirmed in this study. In the results of the National Emphysema Treatment Trial (NETT), it was stated that depression increased the total number of hospitalizations and mortality in sufferers over a three-year period [38]. Treating COPD exacerbations caused depressive symptoms to resolve spontaneously in many patients. The main avenues of treatment are antidepressants and psychotherapy [25,39]. 

Considering that the goal of modern therapeutic procedures is to improve the quality of life of patients with somatic diseases and their comorbidities, research on the quality of life of patients began. Sociodemographic and psychological intercorrelations characterize the environment in which patients live, work, and establish contacts with others in order to ensure the support of the global society to which they belong. Their impact on a patient’s quality of life is still being studied. The results of many studies [38,40,41,42,43] indicate that all categories of health are associated with a lower quality of life and that they affect each other, which was partially confirmed in this study.

Studies testing the relationship between somatic diseases, accompanying comorbidities, and therapeutic medication monitoring have received a lot of attention in recent years. The significance of tracking the relationship between people with pulmonary obstructive disease and the severity of their depression was emphasized in [25]. In fact, this research clarified that the relationship between mMRC and the degree of depression over 8 weeks of depressive disorder treatment is significant. Similarly, concerning the subjects’ quality of life and total life satisfaction (when patients were treated with antidepressants), multiple analyses showed that there was a statistically significant difference among Q-LES-Q-SF scores. 

The study has some limitations and strengths that should be considered. As a limitations, the study sample is modest, which limits the generalization of the results. Nevertheless, it was found that previous studies analyzing the effects of antidepressant treatment on COPD outcomes in COPD patients with depression also had comparatively small sample sizes due to high dropout rates and/or failure to conduct a follow-up with patients for a substantial period of time [39]. Even though this study was conducted on a specific population to examine the association between depression symptoms and the impact of SSRI therapy in COPD subjects on their quality of life appropriately, in addition to observing the effects of SSRIs, it is vital to ascertain the effects of other antidepressant types on COPD participants and to compare the achieved results with a control group. Moreover, there is a lack of research on the association between the COPD level and HAM scores in people with depression, which indicates the need for further research. The mMRC scale was not assessed after 8 weeks of therapy. As noted, the mMRC scale is used to assess dyspnea subjectively. This opens up the possibility for future research, including whether the improvement in the general psychophysical and social functioning after therapy influences the fact that patients perceive their dyspnea as mild, that is, whether it changes to a lower or higher level.

This study also has several strengths. This study focused on patients with COPD and depression simultaneously, excluding other diseases, to ensure the clinical heterogeneity of the study population. Additionally, the study focused only on analyzing the psychophysical and social functioning of patients with COPD and depression comorbidity. The degree of severity of COPD was assessed on the basis of the mMRC scale, which is a measure of subjective feeling, and not via the spirometric degree of airway obstruction (very often, patients with a high mMRC score have mild obstruction seen through spirometry and vice versa) [24]; in this way, we emphasize the connection between psychophysical and social aspects.

## 5. Conclusions

Before treatment, a sizable portion of COPD patients had low mMRC and a high HAM-D1 score. After 8 weeks of therapy, it was discovered that treating COPD-related depression improved depression (HAM-D2). Dyspnea and depressive symptoms are connected, and treating depression may help COPD patients (especially those with severe forms of the disease) manage their psychophysical and social functioning more efficiently. The quality of life and life satisfaction of patients in this study, on average, improved after 8 weeks of SSRI therapy. 

Psychiatrists who treat depressed patients with COPD comorbidity have a simple questionnaire that does not require specific pulmonology knowledge, namely, the mMRC scale, on the basis of which they can, to some extent, predict a therapeutic range of SSRI drugs in this specific group of patients.

Future research should investigate if these results have changed in light of the pandemic and whether people with COPD perceive their overall life satisfaction as worse after the pandemic than before. Concerns and limitations were probably emphasized due to pandemic characteristics and risk groups that needed to be in the strict prevention regimens. It has been shown that it is possible to indicate the benefits of treatment when health personnel identify the onset of depressive symptoms in the early stages of the disease using effective assessment instruments, such as the Hamilton scale.

We suggest that future research include other psychometric scales, as well as investigate the relationships between depressed mood, inflammation, psychopathology, and somatic disorders. Given that the main motive of this research was psychophysical and social functioning in subjects with COPD and depression, the use of multiple prospective designs will be important for future research. An in-depth assessment and understanding of the relationship between depressed mood and the emergence of psychopathology and comorbid disorders would allow for early targeted interventions.

Considering the results of this research, this study can motivate researchers to attribute greater importance to this topic and conduct more research because current psychology is little represented and increasingly important for the patients themselves, particularly in non-highly developed regions. For patients, it is crucial to give great importance to this study and similar future studies to improve their quality of life and overall health outcomes.

To conclude, for the treatment of patients who suffer from both COPD and depression, psychosocial support is considered to be of great significance at all stages of the disease. Hence, we encourage the creation and revision of a series of programs aimed at monitoring patients and their families. A large part of a positive atmosphere is generated by medical workers, because health personnel spend a considerable amount of their time communicating with patients, as well as connecting on a deeper level with both patients and their families. By creating and revising support programs, it would be possible and easier for patients to face the disease, and it would allow medical experts to work with them efficiently. Furthermore, it would be useful in the development of a working atmosphere, which would lead to a higher quality of a patient’s life conditions while facing the disease.

## Figures and Tables

**Table 1:** Demographic, clinical, and social characteristics of the study participants.

Variable	Group	% (N)	Mean	SD	F/Cohen’s d/*p* *
Age	Male	34.84% (30)	47.614	7.311	0.115/−0.489/0.033
	Female	65.52% (57)	51.167	7.206	
Variable	Group	All % (N)	Male % (N)	Female % (N)	χ^2^/*p* **
HAM-D1	1	40.2% (35)	33.3% (10)	43.9% (25)	3.282/0.194
	2	48.3% (42)	46.7% (14)	49.1% (28)	
	3	11.5% (10)	20.0% (6)	7.0% (4)	
HAM-D2	0	37.9% (33)	30.0% (9)	42.1% (24)	4.337/0.261
	1	49.4% (43)	50.0% (15)	49.1% (28)	
	2	12.6% (11)	20.0% (6)	8.8% (5)	
mMRC	1	37.9% (33)	30.0% (9)	42.1% (34)	4.672/0.099
	2	51.7% (45)	50.0% (15)	52.6% (30)	
	3	10.5% (9)	20.0% (6)	5.3% (3)	

* Independent sample *t*-test. ** Chi-squared test for goodness of fit.

**Table 2:** Q-LES-Q-SF scores before and after 8 weeks of therapy according to the degree of dyspnea and gender.

mMRC	Q-LES-Q-SF after	Male			Female		
		Q-LES-Q-SF Scores before 8 Weeks Therapy			Q-LES-Q-SF Scores before 8 Weeks Therapy		
		Bad n (%)	Average * n (%)	Good n (%)	Bad n (%)	Average * n (%)	Good n (%)
1	Average *	5 (35.7)	-	-	14 (50)	3 (1)	-
	Good	-	3 (64.3)	-	2 (6.7)	9 (30)	1 (3.3)
2	Average *	9 (90)	-	-	19 (90.2)	-	-
	Good	-	2 (10)	-	1 (2.5)	4 (7.3)	-
3	Average *	11 (100)	-	-	4 (100)	-	-
	Good	-	-	-	-	-	-

* Neither good nor bad.

**Table 3:** Differences in HAM and Q-LES-Q-SF scores before and after 8 weeks of therapy.

Variable	Mean ± SD	Z/η^2^/*p* *
HAM-D1 score before SSRI therapy	21.79 ± 4.43	−9.528/1.043/ <0.001
HAM-D2 score after 8 weeks of SSRI therapy	13.02 ± 3.97	
Q-LES-Q-SF score before AD	37.30 ± 3.31	−8.191/0.771/ <0.001
Q-LES-Q-SF score after AD	45.07 ± 2.47	
Difference in Q-LES-Q-SF score (after-before)	7.42 ± 1.64	

* Wilcoxon signed-rank test; *p*-value < 0.001.

**Table 4:** Differences in Q-LES-Q-SF domains before and after 8 weeks of therapy.

Study Period	Ability to Function in Daily Life Median (IQR)	Physical Health Median (IQR)	Life Satisfaction Median (IQR)	Relationship to Family Median (IQR)
Baseline	2 (0)	2 (1)	2 (0)	3 (0)
After 8 weeks	3 (0)	3 (1)	3 (0)	3 (1)
Z/η^2^/*p* *	−8.062/0.747/<0.001	−8.442/0.819/<0.001	−8.885/0.908/<0.001	−2.236/0.057/0.060

* Wilcoxon signed-rank test.

**Table 5:** Differences in Q-LES-Q-SF scores and domains before and after 8 weeks of therapy in relation to gender.

Q-LES-Q-SF	Females Median (IQR)	Males Median (IQR)	U/η^2^/*p*
Q-LES-Q-SF score before AD Q-LES-Q-SF score after AD	37 (4)	36 (2)	−2.865/0.675/0.042
	47 (4)	45 (2)	−2.563/0.674/0.040
Life satisfaction before AD Life satisfaction after AD	3 (0)	2 (0)	−3.225/0.676/0.027
	3 (2)	2 (1)	−2.963/0.675/0.036

## Data Availability

Data presented in this study are available from the corresponding author.
